# Integration of an On-Axis General Sun-Tracking Formula in the Algorithm of an Open-Loop Sun-Tracking System

**DOI:** 10.3390/s91007849

**Published:** 2009-09-30

**Authors:** Kok-Keong Chong, Chee-Woon Wong, Fei-Lu Siaw, Tiong-Keat Yew, See-Seng Ng, Meng-Suan Liang, Yun-Seng Lim, Sing-Liong Lau

**Affiliations:** Faculty of Engineering and Science, Universiti Tunku Abdul Rahman,Off Jalan Genting Kelang, Setapak, 53300 Kuala Lumpur, Malaysia; E-Mails: cheewoon_w@hotmail.com (C.-W.W.); jessiesiaw@gmail.com (F.-L.S.); ytkeat@yahoo.com (T.-K.Y.); ngss@utar.edu.my (S.-S.N.); liangms@utar.edu.my (M.-S.L.); yslim@utar.edu.my (Y.-S.L.); lausl@utar.edu.my (S.-L.L.)

**Keywords:** general sun-tracking formula, azimuth-elevation, sun-tracking accuracy, passive sensors, solar concentrator, solar collector

## Abstract

A novel on-axis general sun-tracking formula has been integrated in the algorithm of an open-loop sun-tracking system in order to track the sun accurately and cost effectively. Sun-tracking errors due to installation defects of the 25 m^2^ prototype solar concentrator have been analyzed from recorded solar images with the use of a CCD camera. With the recorded data, misaligned angles from ideal azimuth-elevation axes have been determined and corrected by a straightforward changing of the parameters' values in the general formula of the tracking algorithm to improve the tracking accuracy to 2.99 mrad, which falls below the encoder resolution limit of 4.13 mrad.

## 1. Introduction

Sun-tracking system plays an important role in the development of solar energy applications, especially for the high solar concentration systems that directly convert the solar energy into thermal or electrical energy [1]. Over the past two decades, various types of sun-tracking mechanisms have been proposed to enhance the solar energy harnessing performance of solar collectors. Although the degree of accuracy required depends on the specific characteristics of the solar concentrating system being analyzed, generally the higher the system concentration the higher the tracking accuracy that will be needed [2]. To achieve good tracking accuracy, sun-tracking systems normally employ sensors to feedback error signals to the control system in order to continuously receive maximum solar irradiation on the receiver. The two common types of sensors used for this purpose are closed-loop sensors and open-loop sensors.

Firstly, a closed-loop sensor, such as CCD camera or photo-detector, is used to sense the position of the solar image on the receiver and a feedback signal is sent to the controller if the solar image moves away from the receiver. Sun-tracking systems that employ closed-loop sensors are known as closed-loop sun trackers. Over the past 20 years or so, the closed-loop tracking approach has been traditionally used in the active sun-tracking scheme [3–6]. For example, Kribus *et al.* designed a closed-loop controller for heliostats which improved the pointing error of the solar image up to 0.1 mrad, with the aid of four CCD cameras set on the target [7]. However, this method is rather expensive and complicated because it requires four CCD cameras and four radiometers to be placed on the target. Then the solar images captured by CCD cameras must be analysed by a computer to generate the control correction feedback for correcting tracking errors. In 2006, a sun-tracking error monitoring system that uses a monolithic optoelectronic sensor for a concentrator photovoltaic system was presented by Luque-Heredia *et al.* According to the results from the case study, this monitoring system achieved a tracking accuracy of better than 0.1°. However, the criterion is that this tracking system requires full clear sky days to operate as the incidence light has to be above a certain threshold to ensure that the minimum required resolution is met [8]. That same year, Aiuchi *et al.* developed a heliostat with an equatorial mount and a closed-loop photo-sensor control system. The experimental results showed that the tracking error of the heliostat was estimated to be 2 mrad during fine weather [9]. Nevertheless, this tracking method is not popular and only can be used for sun-trackers with an equatorial mount configuration, which is not a common tracker mechanical structure and is complicated because the central of gravity for the solar collector is far off the pedestal. Furthermore, Chen *et al.* presented studies of digital and analogue sun sensors based on the optical vernier and optical nonlinear compensation measuring principle respectively. The proposed digital and analogue sun sensors have accuracies of 0.02° and 0.2° correspondingly for the entire field of view of ±64° and ±62° respectively [10,11]. The major disadvantage of these sensors is that the field of view, which is in the range of about ±64° for both elevation and azimuth directions, is rather small compared to the dynamic range of motion for a practical sun-tracker that is about ±70° and ±140° for elevation and azimuth directions, respectively. Besides that, it is just implemented at the testing stage in precise sun sensors to measure the position of the sun and has not yet been applied in any closed-loop sun-tracking system so far.

Although closed-loop sun-tracking system can produce a much better tracking accuracy, this type of system will lose its feedback signal when the sensor is shaded or when the sun is blocked by clouds. As an alternative method to overcome the limitation of closed loop sun-trackers, open-loop sun trackers were introduced by using open-loop sensors that do not require any solar image as feedback. The open-loop sensor will ensure that the solar collector is positioned at pre-calculated angles, which are obtained from a special formula or algorithm according to date, time and geographical information. In 2004, Abdallah *et al.* designed a two axes sun tracking system, which is operated by an open-loop control system. A programmable logic controller (PLC) was used to calculate the solar vector and to control the sun tracker so that it follows the sun's trajectory [12]. In addition, Shanmugam *et al.* presented a computer program written in *Visual Basic* that is capable of determining the sun's position and thus drive a paraboloidal dish concentrator (PDS) along the East-West axis or North-South axis for receiving maximum solar radiation [13].

In general, both sun-tracking approaches mentioned above have both strengths and drawbacks, so some hybrid sun-tracking systems have been developed to include both the open-loop and closed-loop sensors. Early in the 21^st^ century, Nuwayhid *et al.* adopted both the open-loop and closed-loop tracking schemes into a parabolic concentrator attached to a polar tracking system. In the open-loop scheme, a computer acts as controller to calculate two rotational angles, i.e., solar declination and hour angles, as well as to drive the concentrator along the declination and polar axes. In the closed-loop scheme, nine light-dependent resistors (LDR) are arranged in an array of a circular-shaped “iris” to facilitate sun-tracking with a high degree of accuracy [14]. In 2006, Luque-Heredia *et al.* proposed a novel *PI* based hybrid sun-tracking algorithm for a concentrator photovoltaic system. In their design, the system can act in both open-loop and closed-loop mode. A mathematical model that involves a time and geographical coordinates function as well as a set of disturbances provides a feedforward open-loop estimation of the sun's position. To determine the sun's position with high precision, a feedback loop was introduced according to the error correction routine which is derived from the estimation of the error of the sun equations that are caused by external disturbances at the present stage based on its historical path [15]. One year later, Rubio *et al.* fabricated and evaluated a new control strategy for a photovoltaic (PV) solar tracker that operated in two tracking modes, i.e., normal tracking mode and search mode. The normal tracking mode combines an open-loop tracking mode that is based on solar movement models and a closed-loop tracking mode which corresponds to the electro-optical controller to obtain a sun-tracking error that is smaller than a specified boundary value and enough for solar radiation to produce electrical energy. Search mode will be started when the sun-tracking error is large or no electrical energy is produced. The solar tracker will move according to a square spiral pattern in the azimuth-elevation plane to sense the sun's position until the tracking error is small enough [16].

As a matter of fact, the tracking accuracy requirement is very much reliant on the design and application of the sun-tracker. In this case, the longer the distance between the solar concentrator and the receiver the higher the tracking accuracy required will be because the solar image becomes more sensitive to the movement of the solar concentrator. As a result, a heliostat or off-axis sun-tracker normally requires much higher tracking accuracy compared to that of on-axis sun-tracker due to the fact that the distance between the heliostat and the target is normally much longer, especially for a central receiver system configuration. In this context, a tracking accuracy in the range of a few miliradians (mrad) is in fact sufficient for an on-axis sun-tracker to maintain its good performance when highly concentrated sunlight is involved [17]. Despite having many existing on-axis sun-tracking methods, the designs available to achieve a good tracking accuracy of a few mrad are complicated and expensive. It is worthwhile to note that conventional on-axis sun-tracking systems normally adopt two common configurations, which are azimuth-elevation and tilt-roll (polar tracking), limited by the available basic mathematical formulas of sun-tracking system. For azimuth-elevation tracking system, the sun-tracking axes must be strictly aligned with both zenith and real north. For a tilt-roll tracking system, the sun-tracking axes must be strictly aligned with both latitude angle and real north. The major cause of sun-tracking errors is how well the aforementioned alignment can be done and any installation or fabrication defects will result in low tracking accuracy. According to our previous study for the azimuth-elevation tracking system, a misalignment of azimuth shaft relative to zenith axis of 0.4° can cause tracking error ranging from 6.45 to 6.52 mrad [18]. In practice, most solar power plants all over the world use a large solar collector area to save on manufacturing cost and this has indirectly made the alignment work of the sun-tracking axes much more difficult. In this case, the alignment of the tracking axes involves an extensive amount of heavy-duty mechanical and civil works due to the requirement for thick shafts to support the movement of a large solar collector, which normally has a total collection area in the range of several tens of square meters to nearly a hundred square meters. Under such tough conditions, a very precise alignment is really a great challenge to the manufacturer because a slight misalignment will result in significant sun-tracking errors. To overcome this problem, an unprecedented on-axis general sun-tracking formula has been proposed to allow the sun-tracker to track the sun in any two arbitrarily orientated tracking axes [18]. In this paper, we would like to introduce a novel sun-tracking system by integrating the general formula into the sun-tracking algorithm so that we can track the sun accurately and cost effectively, even if there is some misalignment from the ideal azimuth-elevation or tilt-roll configuration. In the new tracking system, any misalignment or defect can be rectified without the need for any drastic or labor intensive modifications to either the hardware or software components of the tracking system. In other words, even though the alignments of the azimuth-elevation axes with respect to the zenith-axis and real north are not properly done during the installation, the new sun-tracking algorithm can still accommodate the misalignment by changing the values of *φ*, *λ* and *ζ*࿠࿠ in the tracking program. The advantage of the new tracking algorithm is that it can simplify the fabrication and installation work of solar collectors with higher tolerance in terms of the tracking axes alignment. This strategy has allowed great savings in terms of cost, time and effort by omitting more complicated solutions proposed by other researchers such as adding a closed-loop feedback controller or a flexible and complex mechanical structure to level out the sun-tracking error [1,19]. To demonstrate the use of general formula for improving sun-tracking accuracy, a prototype solar concentrator has been constructed and tested on the campus of Universiti Tunku Abdul Rahman (UTAR).

### 2. Methodology of Using General Formula to Improve Sun-Tracking Accuracy

The derivation of the general formula for an on-axis sun-tracking system has been presented in our previous paper [18]. According to the general formula, the sun-tracking accuracy of the system is highly reliant on the precision of the input parameters of the sun-tracking algorithm: latitude angle (*Φ*), hour angle (*ω*), declination angle (*δ*), as well as the three orientation angles of the tracking axes of solar concentrator, i.e., *φ*, *λ* and *ζ*࿒࿠࿠Among these values, local latitude, *Φ*, and longitude of the sun tracking system can be determined accurately with the latest technology such as a global positioning system (GPS). On the other hand, *ω* and *δ* are both local time dependent parameters (please refer to Appendix for the details). These variables can be computed accurately with the input from precise clock that is synchronized with the Internet time server. As for the three orientation angles *φ*, *λ* and *ζ*࿠, their precision are very much reliant on the care paid during the on-site installation of solar collector, the alignment of tracking axes and the mechanical fabrication. The following mathematical derivation is attempted to obtain analytical solutions for the three orientation angles based on the daily sun-tracking error results induced by the misalignment of sun-tracking axes.

From our previous study [18], the unit vector of the sun, [*S'*], relative to the solar collector can be obtained from a multiplication of four successive coordinate transformation matrices, i.e., [*Φ*], [*φ*], [*λ*] and [*ζ*] with the unit vector of the sun, [*S*], relative to the earth and it is written as:

(1) $$\begin{array}{l} \left[\text{S}\prime \right]=\left[\zeta \right]\left[\lambda \right]\left[\varphi \right]\left[\Phi \right]\left[\text{S}\right],\\ \left[\begin{array}{c} \sin\alpha \\ \cos\alpha \sin\beta \\ \cos\alpha \cos\beta \end{array}\right]=\left[\begin{array}{ccc} \cos\zeta & 0 & \sin\zeta \\ 0 & 1 & 0\\ -\sin\zeta & 0 & \cos\zeta \end{array}\right]\times \left[\begin{array}{ccc} \cos\lambda & -\sin\lambda & 0\\ \sin\lambda & \cos\lambda & 0\\ 0 & 0 & 1 \end{array}\right]\times \left[\begin{array}{ccc} 1 & 0 & 0\\ 0 & \cos\varphi & -\sin\varphi \\ 0 & \sin\varphi & \cos\varphi \end{array}\right]\times \left[\begin{array}{ccc} \cos\Phi & 0 & \sin\Phi \\ 0 & 1 & 0\\ -\sin\Phi & 0 & \cos\Phi \end{array}\right]\times \left[\begin{array}{c} \cos\delta \cos\omega \\ -\cos\delta \sin\omega \\ \sin\delta \end{array}\right], \end{array}$$

where *α* is elevation angle, *β* is azimuth angle, *ω* is hour angle, *δ* is declination angle, *Φ* is latitude at which the solar collector is located as well as *φ*, *λ*࿠ and *ζ* are the three orientation angles of two-orthogonal-driving axes of the solar collector. From the Equation (1), let us multiply the first three transformation matrices [*φ*], [*λ*] and [*ζ*], and then the last two matrices [*Φ*] with [*S*] as to obtain the following result:

(2) $$\begin{array}{l} \left[\begin{array}{c} \sin\alpha \\ \cos\alpha \sin\beta \\ \cos\alpha \cos\beta \end{array}\right]=\left[\begin{array}{ccc} \cos\zeta \cos\lambda & -\cos\zeta \sin\lambda \cos\varphi +\sin\zeta \sin\varphi & \cos\zeta \sin\lambda \sin\varphi +\sin\zeta \cos\varphi \\ \sin\lambda & \cos\lambda \cos\varphi & -\cos\lambda \sin\varphi \\ -\sin\zeta \cos\lambda & \sin\zeta \sin\lambda \cos\varphi +\cos\zeta \sin\varphi & -\sin\zeta \sin\lambda \sin\varphi +\cos\zeta \cos\varphi \end{array}\right]\times \left[\begin{array}{c} \cos\Phi \cos\delta \cos\omega +\sin\Phi \sin\delta \\ -\cos\delta \sin\omega \\ -\sin\Phi \cos\delta \cos\omega +\cos\Phi \sin\delta \end{array}\right]. \end{array}$$

From Equation (2), we can further dissolve it into Equation (3):

(3a) sinα=(cosΦcosδcosω+sinΦsinδ)(cosζcosλ)+(−cosδsinω)(−cosζsinλcosφ+sinζsinφ)+(−sinΦcosδcosω+cosΦsinδ)(cosζsinλsinφ+sinζcosφ)

(3b) cosαsinβ=(cosΦcosδcosω+sinΦsinδ)(sinλ)+(−cosδsinω)(cosλcosφ)+(−sinΦcosδcosω+cosΦsinδ)(−cosλsinφ)

(3c) cosαcosβ=(cosΦcosδcosω+sinΦsinδ)(−sinζcosλ)+(−cosδsinω)(sinζsinλcosφ+cosζsinφ)+(−sinΦcosδcosω+cosΦsinδ)(−sinζsinλsinφ+cosζcosφ)

The time dependency of *ω* and *δ* can be found from Equation (3). Therefore, the instantaneous sun-tracking angles of the collector only vary with the angles *ω* and *δ*. Given three different local times *LCT*_1_, *LCT*_2_ and *LCT*_3_ on the same day, the corresponding three hours angles *ω*_1_, *ω*_2_ and *ω*_3_ as well as three declination angles *δ*_1_, *δ*_2_ and *δ*_3_ can result in three elevation angles *α*_1_, *α*_2_ and *α*_3_ and three azimuth angles *β*_1_, *β*_2_ and *β*_3_ accordingly as expressed in Equation (3a)–(3c). Considering three different local times, we can actually rewrite each of the Equation (3a)–(3c) into three linear equations. By arranging the three linear equations in a matrix form, the Equation (3a)–(3c) can subsequently form the matrices (4a)–(4c):

(4a) $$\begin{array}{l} \left[\begin{array}{c} \sin\alpha_{1}\\ \sin\alpha_{2}\\ \sin\alpha_{3} \end{array}\right]=\left[\begin{array}{ccc} \cos\Phi \cos\delta_{1}\cos\omega_{1}+\sin\Phi \sin\delta_{1} & -\cos\delta_{1}\sin\omega_{1} & -\sin\Phi \cos\delta_{1}\cos\omega_{1}+\cos\Phi \sin\delta_{1}\\ \cos\Phi \cos\delta_{2}\cos\omega_{2}+\sin\Phi \sin\delta_{2} & -\cos\delta_{2}\sin\omega_{2} & -\sin\Phi \cos\delta_{2}\cos\omega_{2}+\cos\Phi \sin\delta_{2}\\ \cos\Phi \cos\delta_{3}\cos\omega_{3}+\sin\Phi \sin\delta_{3} & -\cos\delta_{3}\sin\omega_{3} & -\sin\Phi \cos\delta_{3}\cos\omega_{3}+\cos\Phi \sin\delta_{3} \end{array}\right]\times \left[\begin{array}{c} \cos\zeta \cos\lambda \\ -\cos\zeta \sin\lambda \cos\varphi +\sin\zeta \sin\varphi \\ \cos\zeta \sin\lambda \sin\varphi +\sin\zeta \cos\varphi \end{array}\right]. \end{array}$$

(4b) $$\begin{array}{l} \left[\begin{array}{c} \cos\alpha_{1}\sin\beta_{1}\\ \cos\alpha_{2}\sin\beta_{2}\\ \cos\alpha_{3}\sin\beta_{3} \end{array}\right]=\left[\begin{array}{ccc} \cos\Phi \cos\delta_{1}\cos\omega_{1}+\sin\Phi \sin\delta_{1} & -\cos\delta_{1}\sin\omega_{1} & -\sin\Phi \cos\delta_{1}\cos\omega_{1}+\cos\Phi \sin\delta_{1}\\ \cos\Phi \cos\delta_{2}\cos\omega_{2}+\sin\Phi \sin\delta_{2} & -\cos\delta_{2}\sin\omega_{2} & -\sin\Phi \cos\delta_{2}\cos\omega_{2}+\cos\Phi \sin\delta_{2}\\ \cos\Phi \cos\delta_{3}\cos\omega_{3}+\sin\Phi \sin\delta_{3} & -\cos\delta_{3}\sin\omega_{3} & -\sin\Phi \cos\delta_{3}\cos\omega_{3}+\cos\Phi \sin\delta_{3} \end{array}\right]\times \left[\begin{array}{c} \sin\lambda \\ \cos\lambda \cos\varphi \\ -\cos\lambda \sin\varphi \end{array}\right]. \end{array}$$

(4c) $$\begin{array}{l} \left[\begin{array}{c} \cos\alpha_{1}\cos\beta_{1}\\ \cos\alpha_{2}\cos\beta_{2}\\ \cos\alpha_{3}\cos\beta_{3} \end{array}\right]=\left[\begin{array}{ccc} \cos\Phi \cos\delta_{1}\cos\omega_{1}+\sin\Phi \sin\delta_{1} & -\cos\delta_{1}\sin\omega_{1} & -\sin\Phi \cos\delta_{1}\cos\omega_{1}+\cos\Phi \sin\delta_{1}\\ \cos\Phi \cos\delta_{2}\cos\omega_{2}+\sin\Phi \sin\delta_{2} & -\cos\delta_{2}\sin\omega_{2} & -\sin\Phi \cos\delta_{2}\cos\omega_{2}+\cos\Phi \sin\delta_{2}\\ \cos\Phi \cos\delta_{3}\cos\omega_{3}+\sin\Phi \sin\delta_{3} & -\cos\delta_{3}\sin\omega_{3} & -\sin\Phi \cos\delta_{3}\cos\omega_{3}+\cos\Phi \sin\delta_{3} \end{array}\right]\times \left[\begin{array}{c} -\sin\zeta \cos\lambda \\ \sin\zeta \sin\lambda \cos\varphi +\cos\zeta \sin\varphi \\ -\sin\zeta \sin\lambda \sin\varphi +\cos\zeta \cos\varphi \end{array}\right]. \end{array}$$

where the angles *Φ*, *φ*, *λ*࿠ and *ζ*࿠are constants with respect to the local time.

In practice, we can measure the sun tracking angles, i.e., (*α*_1_, *α*࿠_2_, *α*_3_) and (*β*_1_, *β*_2_, *β*_3_) during sun-tracking at three different local times via a recorded solar image of the target using a CCD camera. With the recorded data, we can compute the three arbitrary orientation angles *φ*, *λ* and *ζ*࿠of the solar collector using the third-order determinants method to solve the three simultaneous equations as shown in Equation (4a)–(4c). From Equation (4b), the orientation angle *λ* can be determined as follows:

(5a) $$\lambda =\sin^{-1}\left(\frac{\left|\begin{array}{ccc} \cos\alpha_{1}\sin\beta_{1} & -\cos\delta_{1}\sin\omega_{1} & -\sin\Phi \cos\delta_{1}\cos\omega_{1}+\cos\Phi \sin\delta_{1}\\ \cos\alpha_{2}\sin\beta_{2} & -\cos\delta_{2}\sin\omega_{2} & -\sin\Phi \cos\delta_{2}\cos\omega_{2}+\cos\Phi \sin\delta_{2}\\ \cos\alpha_{3}\sin\beta_{3} & -\cos\delta_{3}\sin\omega_{3} & -\sin\Phi \cos\delta_{3}\cos\omega_{3}+\cos\Phi \sin\delta_{3} \end{array}\right|}{\left|\begin{array}{ccc} \cos\Phi \cos\delta_{1}\cos\omega_{1}+\sin\Phi \sin\delta_{1} & -\cos\delta_{1}\sin\omega_{1} & -\sin\Phi \cos\delta_{1}\cos\omega_{1}+\cos\Phi \sin\delta_{1}\\ \cos\Phi \cos\delta_{2}\cos\omega_{2}+\sin\Phi \sin\delta_{2} & -\cos\delta_{2}\sin\omega_{2} & -\sin\Phi \cos\delta_{2}\cos\omega_{2}+\cos\Phi \sin\delta_{2}\\ \cos\Phi \cos\delta_{3}\cos\omega_{3}+\sin\Phi \sin\delta_{3} & -\cos\delta_{3}\sin\omega_{3} & -\sin\Phi \cos\delta_{3}\cos\omega_{3}+\cos\Phi \sin\delta_{3} \end{array}\right|}\right)$$

Similarly, the other two remaining orientation angles, *φ* and *ζ* can be resolved from Equation (4b) and Equation (4c) respectively as follows:

(5b) $$\varphi =\sin^{-1}\left(\frac{\left|\begin{array}{ccc} \cos\Phi \cos\delta_{1}\cos\omega_{1}+\sin\Phi \sin\delta_{1} & -\cos\delta_{1}\sin\omega_{1} & \cos\alpha_{1}\sin\beta_{1}\\ \cos\Phi \cos\delta_{2}\cos\omega_{2}+\sin\Phi \sin\delta_{2} & -\cos\delta_{2}\sin\omega_{2} & \cos\alpha_{2}\sin\beta_{2}\\ \cos\Phi \cos\delta_{3}\cos\omega_{3}+\sin\Phi \sin\delta_{3} & -\cos\delta_{3}\sin\omega_{3} & \cos\alpha_{3}\sin\beta_{3} \end{array}\right|}{\left|\begin{array}{ccc} \cos\Phi \cos\delta_{1}\cos\omega_{1}+\sin\Phi \sin\delta_{1} & -\cos\delta_{1}\sin\omega_{1} & -\sin\Phi \cos\delta_{1}\cos\omega_{1}+\cos\Phi \sin\delta_{1}\\ \cos\Phi \cos\delta_{2}\cos\omega_{2}+\sin\Phi \sin\delta_{2} & -\cos\delta_{2}\sin\omega_{2} & -\sin\Phi \cos\delta_{2}\cos\omega_{2}+\cos\Phi \sin\delta_{2}\\ \cos\Phi \cos\delta_{3}\cos\omega_{3}+\sin\Phi \sin\delta_{3} & -\cos\delta_{3}\sin\omega_{3} & -\sin\Phi \cos\delta_{3}\cos\omega_{3}+\cos\Phi \sin\delta_{3} \end{array}\right|}\frac{1}{-\cos\lambda }\right)$$

(5c) $$\zeta =-\sin^{-1}\left(\frac{\left|\begin{array}{ccc} \cos\alpha_{1}\cos\beta_{1} & -\cos\delta_{1}\sin\omega_{1} & -\sin\Phi \cos\delta_{1}\cos\omega_{1}+\cos\Phi \sin\delta_{1}\\ \cos\alpha_{2}\cos\beta_{2} & -\cos\delta_{2}\sin\omega_{2} & -\sin\Phi \cos\delta_{2}\cos\omega_{2}+\cos\Phi \sin\delta_{2}\\ \cos\alpha_{3}\cos\beta_{3} & -\cos\delta_{3}\sin\omega_{3} & -\sin\Phi \cos\delta_{3}\cos\omega_{3}+\cos\Phi \sin\delta_{3} \end{array}\right|}{\left|\begin{array}{ccc} \cos\Phi \cos\delta_{1}\cos\omega_{1}+\sin\Phi \sin\delta_{1} & -\cos\delta_{1}\sin\omega_{1} & -\sin\Phi \cos\delta_{1}\cos\omega_{1}+\cos\Phi \sin\delta_{1}\\ \cos\Phi \cos\delta_{2}\cos\omega_{2}+\sin\Phi \sin\delta_{2} & -\cos\delta_{2}\sin\omega_{2} & -\sin\Phi \cos\delta_{2}\cos\omega_{2}+\cos\Phi \sin\delta_{2}\\ \cos\Phi \cos\delta_{3}\cos\omega_{3}+\sin\Phi \sin\delta_{3} & -\cos\delta_{3}\sin\omega_{3} & -\sin\Phi \cos\delta_{3}\cos\omega_{3}+\cos\Phi \sin\delta_{3} \end{array}\right|}\times \frac{1}{\cos\lambda }\right)$$

**Figure 1.**
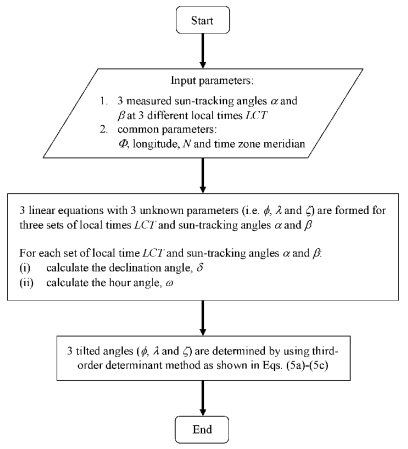
The flow chart of the computational program to determine the three unknown orientation angles that cannot be precisely measured by tools in practice, i.e., *φ*, *λ* and *ζ*.

Figure 1 shows the flow chart of the computational program designed to solve the three unknown orientation angles of the solar collector: *φ*, *λ*࿠ and *ζ*࿠using Equation (5a)–(5c). By providing the three sets of actual sun tracking angles࿔ *α*࿠and࿠*β*࿔*࿠*at different local times for a particular number of day as well as geographical information, i.e., longitude and latitude, the computational program can be executed to calculate the three unknown orientation angles.

## 3. Open-Loop Sun-Tracking System Design

**Figure 2.**
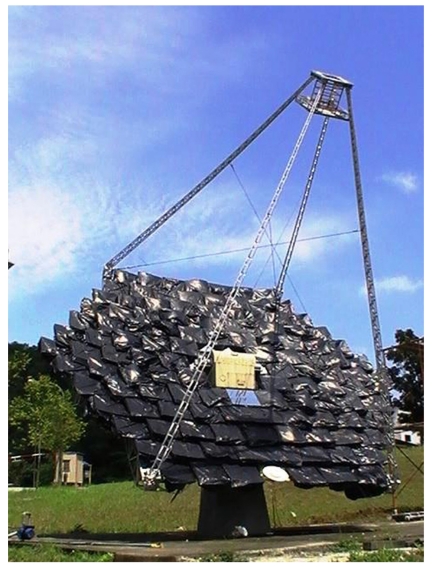
A prototype of on-axis solar concentrator that has been constructed at Universiti Tunku Abdul Rahman (UTAR).

To test the aforementioned methodology, a prototype of an on-axis solar concentrator with a total reflective area of 25 m^2^ has been constructed in the campus of UTAR, Kuala Lumpur (located at latitude 3.22° and longitude 101.73°; see Figure 2). The prototype consists of 120 sets of mirrors that are arranged into a hexagonal array and the target is placed at a focal point with the distance of 4.5 m from the centre of solar concentrator frame. This solar concentrator is designed to operate on the most common two-axis tracking system, which is the azimuth-elevation tracking system. The drive mechanism for the solar concentrator consists of stepper motors and associated gears. Two stepper motors, with 0.72 degree in full step, are coupled to the shafts, elevation and azimuth shafts, with a gear ratio of 4,400 yielding an overall resolution of 1.64 × 10^–4^°/step.

**Figure 3.**
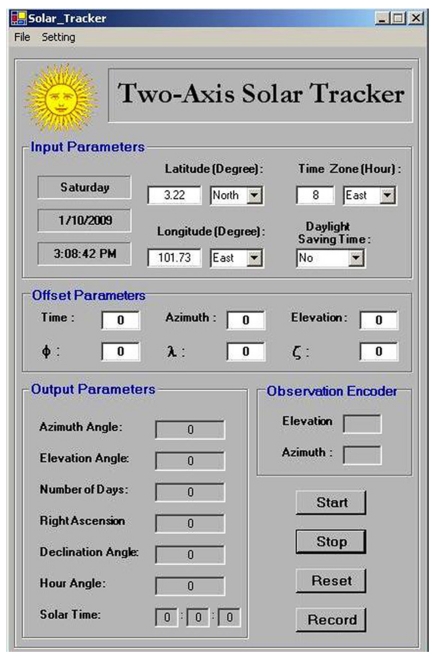
A Windows-based control program that has been integrated with the on-axis general formula.

A Windows-based control program has been developed by integrating the general formula into the open-loop sun-tracking algorithm. In the control algorithm, the sun-tracking angles, i.e., azimuth (*β* and elevation (*α*࿗ angles, are first computed according to the latitude (*Φ*࿗, longitude, day numbers (*N*), local time (*LCT*), and the three orientation angles *φ*, *λ* and *ζ*. The control program then generates digital pulses that are sent to the stepper motor to drive the concentrator to the pre-calculated angles along azimuth and elevation movements in sequence. Each time, the control program only activates one of the two stepper motors through a relay switch. The executed control program of sun-tracking system is shown in Figure 3.

**Figure 4.**
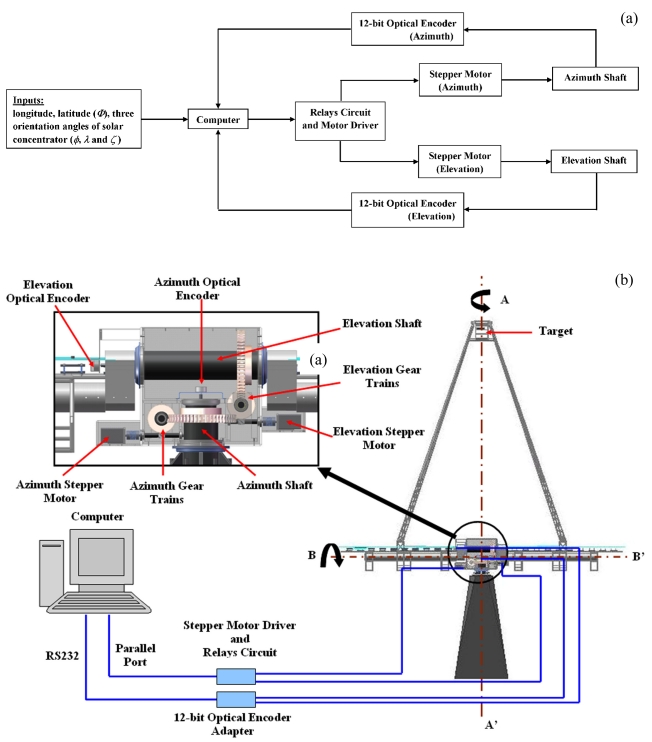
**(a).** Block diagram to show the complete open-loop feedback system of the prototype solar concentrator. **(b).** Schematic diagram to show the detail of the open-loop sun-tracking system of the prototype solar concentrator where AA' is azimuth-axis and BB' is elevation-axis.

An open-loop control system is preferable for the prototype solar concentrator so as to keep the design of the sun-tracker simple and cost effective. In our design, open-loop sensors, 12-bit absolute optical encoders with a precision of 2,048 counts per revolution, are attached to the shafts along the azimuth and elevation axes of the concentrator to monitor the turning angles and to send feedback signals to the computer if there is any abrupt change in the encoder reading [see the inset of Figure 4(b)]. Therefore, the sensors not only ensure that the instantaneous azimuth and elevation angles are matched with the calculated values from the general formula, but also eliminate any tracking errors due to mechanical backlash, accumulated error, wind effects and other disturbances to the solar concentrator. With the optical encoders, any discrepancy between the calculated angles and real time angles of solar concentrator can be detected, whereby the drive mechanism will be activated to move the solar concentrator to the correct position. The block diagram and schematic diagram for the complete design of the open-loop control system of the prototype are shown in Figure 4 (a),(b), respectively.(a)

The estimated total electrical energy produced by the prototype solar concentrator and the total energy consumption of the sun-tracking system are also calculated. Taking into account the total mirror area of 25 m^2^, optical efficiency of 85%, and the conversion efficiency from solar energy to electrical energy of 30% for direct solar irradiation of 800 W/m^2^, we have obtained a generated output energy of 35.7 kW/h/day for seven hours of daily sunshine. Table 1 shows the energy consumption of 1.26 kW/h/day for the prototype includes the tracking motors, motor driver, encoders and computer. It corresponds to less than 3.5% of the rated generated output energy. Among all these components, the computer consumes the most power (more than 100 W) and in the future a microcontroller could be used to replace computer as to reduce the energy consumption.

## 4. Performance Study and Experimental Results

**Figure 5.**
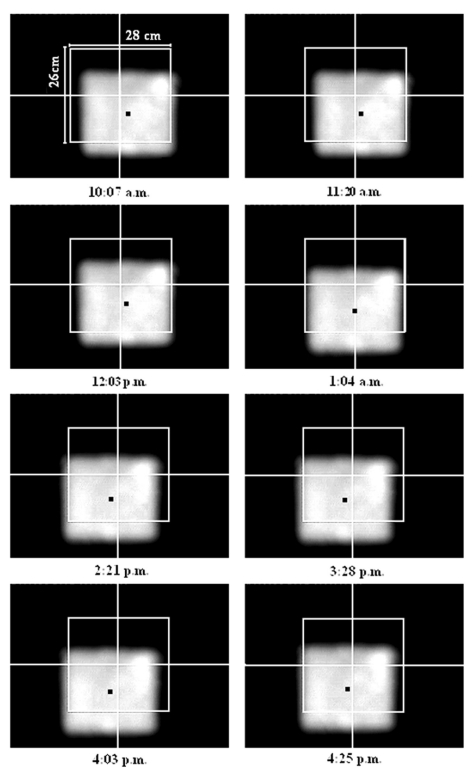
The recorded solar images on the target of prototype solar concentrator using a CCD camera from 10:07 am. to 4:25 pm. on 13 January 2009 with *φ*࿠= *λ*࿠= *ζ*࿠= 0°.

**Figure 6.**
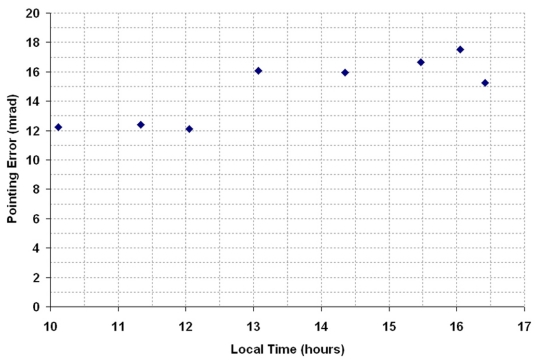
The plot of pointing error (mrad) versus local time (hours) for the parameters, i.e.,࿠*φ*࿠= *λ*࿠= *ζ*࿠= 0°, on 13 January 2009.

Before the performance of the sun-tracking system was tested, 119 sets of mirrors were covered with black plastic, except the one mirror which is located nearest to the centre of the concentrator frame so that we can analyse the tracking accuracy by only observing the movement of one solar image at the target. To avoid the sun-tracking errors due to wrong estimation of the prototype's geographical location, a GPS was used to determine the latitude (*Φ*) and longitude of the solar concentrator. Initially, we assume that the alignment of solar concentrator is perfectly done relative to real north and zenith by setting the three orientation angles as *φ*࿠= *λ*࿠= *ζ࿠*= 0° in the control program. To study the performance of sun-tracking system on 13 January 2009, a CCD camera was employed to capture the solar image cast on the target for every 30 minutes from 10 am. to 5 pm. local time. A CCD camera with 640 × 480 pixels resolution was connected to a computer via a PCI video card to have a real time transmission and recording of solar image. Figure 5 illustrates some of the recorded solar images at different local times. According to the recorded results shown in Figure 6, the recorded tracking errors, ranging from 12.12 to 17.54 mrad throughout the day, have confirmed that the solar concentrator is misaligned relative to zenith and real north.

**Figure 7.**
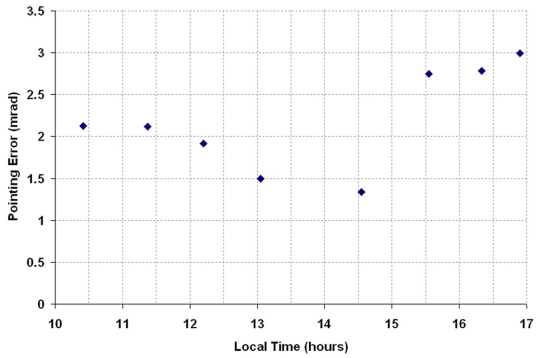
The plot of pointing error (mrad) versus local time (hours) for the parameters, i.e.,࿠*φ*࿠= −0.1°; *λ*࿠= 0°; and *ζ*࿠= −0.5°, on 16 January 2009.

**Figure 8.**
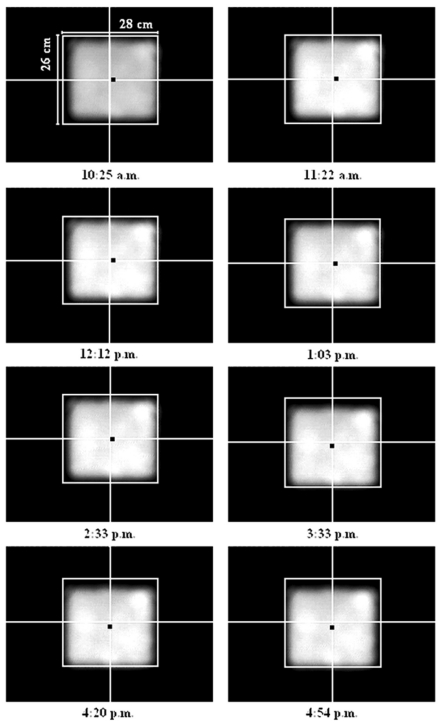
The recorded solar images on the target of prototype solar concentrator using a CCD camera from 10:25 am. to 4:54 pm. on 16 January 2009 with *φ*࿠= −0.1°; *λ*࿠= 0°; and *ζ*࿠= −0.5°.

To rectify the problem of the sun-tracking errors due to imperfect alignment of the solar concentrator during the installation, we have to determine the three misaligned angles, i.e., *φ*, *λ*࿔ *ζ*, and then insert these values into the edit boxes provided by the control program as shown in Figure 3. Thus, the computational program using the methodology as described in Figure 1 was executed to compute the three new orientation angles of the prototype based on the data captured on 13 January 2009. The actual sun-tracking angles, i.e., *α* and *β*, can be determined from the solar image position relative to the central point of the target. Three sets of sun-tracking angles at three different local times from the previous data were used as the input values to the computational program for simulating the three unknown parameters of *φ*, *λ*࿠ and *ζ*࿒ The simulated results are *φ*࿠= −0.1°, *λ*࿠= 0°, and *ζ*࿠= −0.5°. To substantiate the simulated results, these values were then used in the next session of sun-tracking that was performed on 16 January 2009 from 10 am. to 5 pm. With the new orientation angles, the performance of the prototype in sun-tracking has been successfully improved to the accuracy of 2.99 mrad, as shown in Figure 7. This result has reached the limit of sun-tracking accuracy due to the resolution of the optical encoder which corresponds to 4.13 mrad, unless higher resolution optical encoders are used as sensors. Figure 8 shows the recorded solar images at the target for different local times ranging from 10 am. to 5 pm. on 16 January 2009. In the experimental results, even though the misalignment on the azimuth axis is in the range of 0.5°, the resulted sun-tracking error is significant, especially for the application in high concentration solar collectors and in particular for dense array concentrator photovoltaic systems [17]. Since then, the prototype has been tested by running it for a period of more than six months to confirm the validation of the sun-tracking results.

## 5. Conclusions

With the simulated parameter *φ*࿠= −0.1°; *λ*࿠= 0°; and *ζ*࿠= −0.5°, the performance of a prototype in sun-tracking has been improved to a maximum pointing error of 2.99 mrad, which falls below the encoder resolution, 4.13 mrad. As a result, the general sun-tracking formula is confirmed to be capable of rectifying the installation error of the solar concentrator with a significant improvement in the tracking accuracy. In fact, there are many solutions of improving the tracking accuracy such as adding a closed-loop feedback system to the controller [1], designing a flexible mechanical platform capable of two-degree-of-freedom for fine adjustment of azimuth shaft [19], etc. Nevertheless, all these solutions require a more complicated sun tracker engineering designs, which also make the whole system more costly. Instead of using a complicated sun-tracking method, integration of an on-axis general sun-tracking formula into the open-loop sun-tracking system is a clever method of obtaining a reasonably high precision in sun-tracking with a simple and cost effective design. This approach can significantly improve the performance and reduce the cost of solar energy collectors, especially for high concentration systems.

## Figures and Tables

**Table 1.** Specification and energy consumption of prototype sun-tracking system.

Total rotational angles of Elevation axis (degree/ day)	240
Total rotational angles of Azimuth axis (degree/ day)	540
Motor's rotational speed (rpm)	120
Gear ratio	1: 4,400
Solar concentrator's angular speed (degree per second)	0.16
Total time for Elevation axis rotation (hour/ day)	0.41
Total time for Azimuth axis rotation (hour/ day)	0.92
Total operating time:10am–5pm (hour/ day)	7
	
Elevation motor's power consumption (watt)	99
Azimuth motor's power consumption (watt)	66
Power consumption of computer, encoders & motor driver (watt)	165
	
Energy Consumption of the Elevation motor (kW-h/day)	0.04
Energy Consumption of the Azimuth motor (kW-h/day)	0.06
Energy Consumption of computer, encoder & driver (kW-h/day)	1.16
	
Total Energy Consumption of the motors (kW-h/day)	1.26

## Appendix

Formula for declination angle:

sinδ=0.39795cos[0.98563(N−173)]

where *N* is day number and calendar dates are expressed as the *N* = 1, starting with January 1. Thus March 22 would be *N* = 31 + 28 + 22 = 81 and December 31 means *N* = 365.

The hour angle expresses the time of day with respect to the solar noon: It is the angle between the plane of the meridian containing observer and meridian that touches the earth-sun line. It is zero at solar noon and increases by 15° every hour:

$$\omega =15(t_{s}-12)\;(\mathrm{degree})$$

where *t_s_* is the solar time in hours. A solar time is a 24-hour clock with 12:00 as the exact time when the sun is at the highest point in the sky. The concept of solar time is to predict the direction of the sun's ray relative to a point on the earth. Solar time is location or longitudinal dependent. It is generally different from local clock time (*LCT*) (defined by politically time zones)

The conversion between solar time and local clock time requires knowledge of the location, the day of the year, and the standards to which local clocks are set. The conversion between solar time *t_s_* and local clock time (*LCT*) (in 24-hour rather than AM/PM format) takes the form:

$$t_{s}=\mathrm{LCT}+\mathrm{EOT}/60-LC-D\;(\mathrm{hours})$$

where *EOT* is the equation of time in minutes, *LC* is a longitude correction, and *D* is daylight saving time. Daylight saving time was initiated in the spring of 1918 to “save fuel and promote other economies in a country at war” [20]. According to this concept, the standard time is advanced by 1 hour, usually from 2:00 am. on the last Sunday in April until 2:00 am. on the last Sunday in October.

The difference between mean time and solar time is called the equation of time (*EOT*). An approximation for the equation of time in minutes is given by Woolf (1968) is accurate to within about 30 seconds during daylight hours:

EOT=0.258cosx−7.416sinx−3.648cos2x−9.228sin2x(miutes)

where the angle *x* is a function of the day number *N*,

x=360(N−1)/365.242(degrees)

For example, on February 11, 1981 the day number *N* = 42, *x* = 40.41 degrees and *EOT* = −14.35 minutes. Formula for longitudinal correction:

LC=(locallongitude−longitude of Standard time zone meridian)/15(hours)
